# The complete mitochondrial genome of *Eclipophleps carinata* (Orthoptera: Acridoidea: Gomphoceridae)

**DOI:** 10.1080/23802359.2021.1907803

**Published:** 2021-04-01

**Authors:** Hongge Qian, Dorjsuren Altanchimeg, Non Naizab, Shusen Wang, Suyaletu Wen, Chen Lin

**Affiliations:** a Institute of Life Science and Technology, Inner Mongolia Normal University, Hohhot, China; b College of Bioscience and Biotechnology, Hunan Agricultural University, Changsha, China; c Institute of Biology, Mongolian Academy of Sciences, Ulaanbaatar, Mongolia; d College of Desert Control Science and Engineering, Inner Mongolia Agricultural University, Hohhot, China; e Wulatehou Banner Forestry Bureau, Bayannaoer, China; f College of Plant Protection, China Agricultural University, Beijing, China

**Keywords:** Mitochondrial genome, *Eclipophleps carinata*, Gomphoceridae

## Abstract

The complete *Eclipophleps carinata* mitogenome was sequenced through Illumina HiSeq 2500 platform and the resulting data were analyzed in this paper. The mitochondrial genome of *E. carinata* is a typical circular DNA molecule of 15,781 bp with 37 genes and 74.5% A + T content, which encoded 13 protein-coding genes (PCGs), 22 tRNA genes, two rRNA genes, and the control region. The *E. carinata* mitochondrial genome and 27 mitochondrial genomes (downloaded from NCBI) were employed to construct phylogenetic tree, in which *Ruspolia dubia* and *Teleogryllus occipitails* were the outgroups. Phylogenetic reconstruction validated the taxonomic status of *E. carinata*, which was placed in the monophyletic Gomphocerinae in Acrididae.

*Eclipophleps carinata* L. Mitshenko, 1968 belongs to *Eclipophleps* using morphometrics. It is only found in the high alpine meadows area of western Mongolia. The genetics of *E. carinata* have not been investigated despite the interest in studying grasshopper biodiversity of western Mongolia. Here, we present the complete mitochondrial genome of the adult *E. carinata* collected from Mongolia in July 2019, and the typical specimen was kept in the Inner Mongolia Normal University nature museum.

## Materials and methods

The specimens in this experiment were taxonomically identified by Professor Nonnaizab from Inner Mongolia Normal University, and the *E. carinata* specimen was collected from Mongolia, Lake Tonkhil, Tonkhil soum, province Govi Altai (46.15131 N, 93.53942 E, 2107) in July 2019.

The total genomic DNA was extracted and sequenced using Illumina HiSeq 2500 platform, de novo assembly was conducted by SPAdes v3.10.1 (http://cab.spbu.ru/software/spades/) with K-mer auto (Boardman et al. 2019).

Different from the published mitochondrial genomic of the Gomphoceridae in NCBI, the location and fragment size of protein-coding genes, tRNA genes, rRNA genes and D-loop region were determined. tRNAscan-search server (http://lowelab.ucsc.edu/tRNAscan-SE/) and MITOS web server (http://mitos.bioinf.uni-leipzig.de/index.py) were applied to predict the tRNA genes secondary structure, which cannot be predicted accurately on the online website, thus it is necessary to manually predict the secondary structure of tRNA gene according to the published secondary structure of tRNA gene. The base content, sequence length and codon preference of mitochondrial genome, J-chain coding genes, N-chain coding genes were analyzed and counted in MEGA X respectively (Sudhir et al. 2018).

We retrieved and downloaded nucleotide sequences of the 13 protein-coding genes for 27 species of insects from the NCBI. Protein-coding genes of the *E. carinata* determined in the current study were added, thus generating a dataset of 28 taxa (27 Acrididae species and two outgroup species (*Ruspolia dubia* and *Teleogryllus occipitails* as outgroups)). Each protein-coding gene was aligned individually based on codon-based multiple alignments using the MAFFT (Kazutaka et al. 2005) algorithm within the Phylosuite (Zhang et al. 2020). Poorly aligned sites were removed from the protein alignment before back-translate to nucleotides. The best‐fit evolutionary model was searched by ModelFinder (Kalyaanamoorthy et al. 2017) algorithm within the Phylosuite. The ML phylogenetic analyses were performed by IQ-TREE v1.6.8 (Nguyen et al. 2015), select Ultrafast for ‘Bootstrap,’ select 5000 for ‘Num of Bootstrap,’ ensure SH-alRT test, and the default value of repeated sampling is 1000.

## Results

The mitochondrial genome of *E. carinata* is a typical circular DNA molecule of 15,781 bp with 37 genes and biased toward A and T, with 74.4%, it included 42.9% A, 31.5% T, 15.0% C, and 10.6% G, submitted to NCBI (https://www.ncbi.nlm.nih.gov/genbank/), which was assigned the accession MN968347.

The location of the 13 PCGs is identical to that for *Gomphocerippus rufus* mitogenome with nine PCGs on the J chain, and four on the N chain (Sun et al. 2010). The A + T content of protein-coding genes, tRNA genes, rRNA genes, and control region are all higher than that of G + C, which are 73.8%, 72.2%, 76.0%, and 79.6% respectively.

Among the PCGs, ATG is the start codon among seven PCGs (*ND2, COII, ATP6, COIII, ND4, ND4L,* and *CYTB*), ATT for three (*ND5*, *ND6,* and *COI*), ATA for two (*ND3* and *ND1*), and ATC for *ATP8*, TAA is the stop codon for 12 PCGs and TAG for *ND1*. The overlapping areas of *ATP6/ATP8* and *ND4/ND4L* are both 7 bp, which is ‘ATGATAA,’ this phenomenon was also found in many invertebrates, including *Brachyrhynchus hsiaoi* (Li et al. 2016)*, Aradus compar*, *Libiocoris heissi*, and *Aneurus sublobatus* (Song et al. 2016).

The mitogenome consisted of 22 tRNAs and two rRNAs. All of 22 tRNAs range from 62 bp (*tRNA^Pro^*) to 71 bp(*tRNA^Val^*). The DHU arm of *tRNA^Ser(AGN)^* has only one base pair and can not form a typical secondary structure, there are only two bases on the DHU loop. Both rRNAs are AT rich with >70%. The *16S rRNA* is 1302 bp and the *12S rRNA* is 792 bp. The length of the control region is 948 bp.

**Figure 1.**
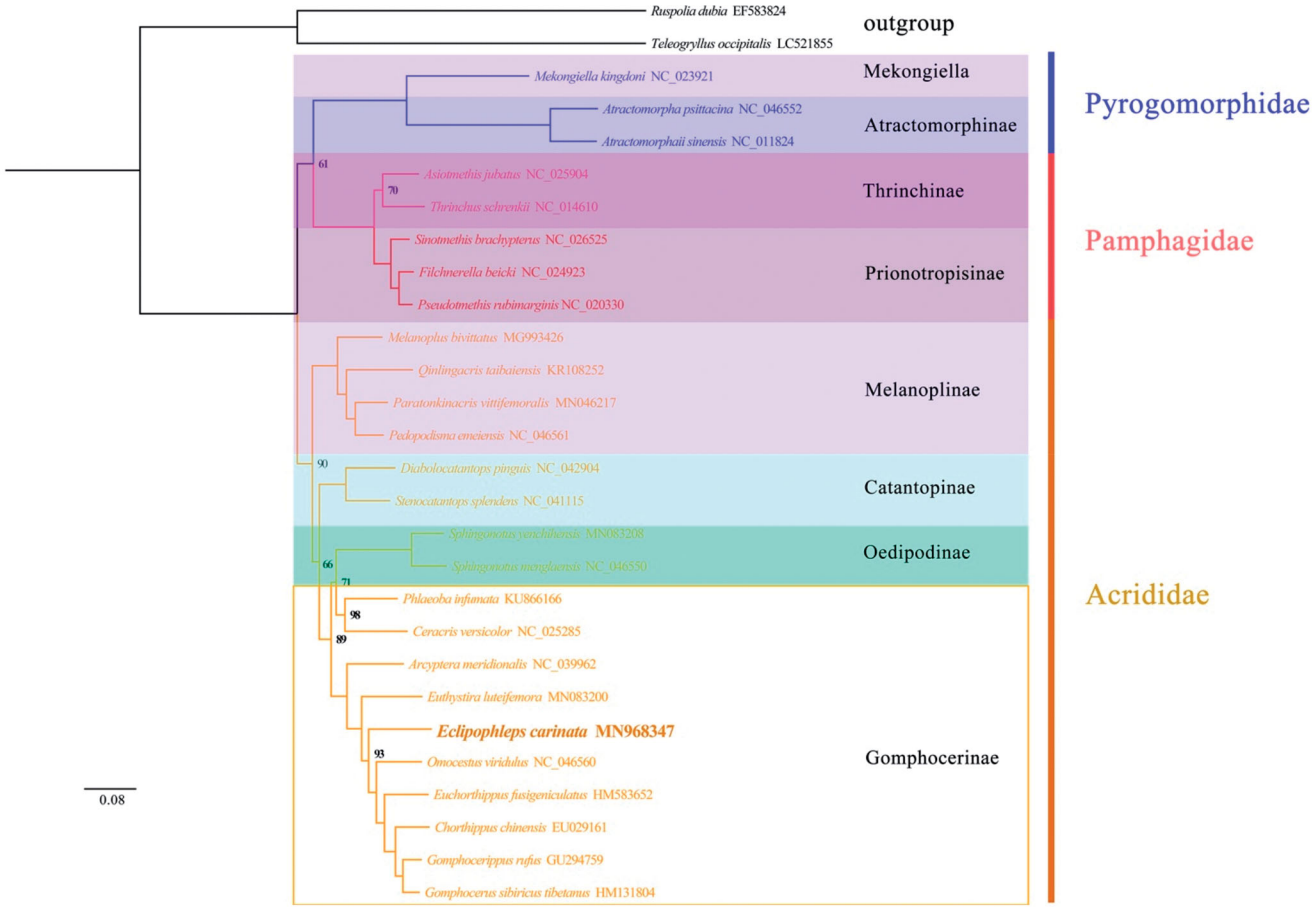
Phylogenetic tree obtained from maximum-likelihood (ML) analysis based on PCGs of 28 species. The best‐fit evolutionary model is GTR + F+G4.

We constructed the maximum-likelihood (ML) phylogenetic tree based on the 13 PCGs of 28 species (including two outgroups from Gryllidae and Tettigoniidae). The phylogenetic tree topology structure is (Pyrgomorphidae+ (pamphagidae+ (Acrididae))) (Figure 1). *Eclipophleps carinata* is assigned to be within the family of Gomphocerinae in Acridoidea. The phylogenetic relationship resulted from this study bearing bothsimilarities and differences in comparison to those based on morphology. Except for Gomphocerinae, all taxa in this study are monophyletic, which is supported by very high-confidence value in both trees. It is obvious that dense taxonomic sampling of Gomphocerinae is in demand to analyze the phylogenetic position of this group of grasshoppers (Zhang et al. 2016).

## Data Availability

The genome sequence data that support the findings of this study are openly available in GenBank of NCBI at (https://www.ncbi.nlm.nih.gov/) under the accession no. MN968347. The associated BioProject, SRA, and Bio-Sample numbers are PRJNA685974, SRR13269645, and SAMN17102428 respectively.

## References

[CIT0001] Boardman L, Eimanifar A, Kimball R, Braun E, Fuchs S, Grünewald B, Ellis JD. 2019. The mitochondrial genome of *Apis mellifera simensis* (Hymenoptera: Apidae), an Ethiopian honey bee. Mitochondrial DNA B Resour. 5(1):9–10. 33366397 10.1080/23802359.2019.1693307 PMC7721028

[CIT0002] Kalyaanamoorthy S, Minh BQ, Wong TKF, von Haeseler A, Jermiin LS. 2017. ModelFinder: fast model selection for accurate phylogenetic estimates. Nat Methods. 14(6):587–589. 28481363 10.1038/nmeth.4285 PMC5453245

[CIT0003] Kazutaka K. 2005. MAFFT version 5: improvement in accuracy of multiple sequence alignment. Nucleic Acids Res. 33(2):511–518. 15661851 10.1093/nar/gki198 PMC548345

[CIT0004] Li H, Shi A, Song F, Cai W. 2016. Complete mitochondrial genome of the flat bug *Brachyrhynchus hsiaoi* (Hemiptera: Aradidae). Mitochondrial DNA A DNA Mapp Seq Anal. 27(1):14–15. 24438289 10.3109/19401736.2013.867437

[CIT0005] Nguyen L-T, Schmidt HA, von Haeseler A, Minh BQ. 2015. IQ-TREE: a fast and effective stochastic algorithm for estimating maximum-likelihood phylogenies. Mol Biol Evol. 32(1):268–274. 25371430 10.1093/molbev/msu300 PMC4271533

[CIT0006] Song F, Li H, Shao R, Shi A, Bai X, Zheng X, Heiss E, Cai W. 2016. Rearrangement of mitochondrial tRNA genes in flat bugs (Hemiptera: Aradidae). Entific Rep. 6:25725. 10.1038/srep25725 PMC4867608 27180804

[CIT0007] Sudhir K, Glen S, Michael L, Knyaz C, Tamura K. 2018. MEGA X: molecular evolutionary genetics analysis across computing platforms. Mol Biol Evol. 35(6):1547–1549. 29722887 10.1093/molbev/msy096 PMC5967553

[CIT0008] Sun H, Zheng Z, Huang Y. 2010. Sequence and phylogenetic analysis of complete mitochondrial DNA genomes of two grasshopper species *Gomphocerus rufus* (Linnaeus, 1758) and *Primnoa arctica* (Zhang and Jin, 1985) (Orthoptera: Acridoidea). Mitochondr DNA. 21(3–4):115–131. 10.3109/19401736.2010.482585 10.3109/19401736.2010.482585 20482331

[CIT0009] Zhang D, Gao F, Jakovlić I, Zou H, Zhang J, Li WX, Wang GT. 2020. Phylosuite: an integrated and scalable desktop platform for streamlined molecular sequence data management and evolutionary phylogenetics studies. Mol Ecol Resour. 20(1):348–355. 31599058 10.1111/1755-0998.13096

[CIT0010] Zhang Y, Liu B, Zhang H, Yin H, Zhang D. 2016. The complete mitochondrial genome of *Pacris xizangensis* (Orthoptera: Acridoidea: Gomphoceridae). Mitochondrial DNA A DNA Mapp Seq Anal. 27(1):320–321. 24617470 10.3109/19401736.2014.892097

